# Extreme Genetic Structure in a Small-Bodied Freshwater Fish, the Purple Spotted Gudgeon, *Mogurnda adspersa* (Eleotridae)

**DOI:** 10.1371/journal.pone.0040546

**Published:** 2012-07-12

**Authors:** Jane M. Hughes, Kathryn M. Real, Jonathan C. Marshall, Daniel J. Schmidt

**Affiliations:** 1 Australian Rivers Institute, Griffith University, Brisbane, Australia; 2 Environment and Resource Sciences Division, Queensland Department of Science, IT, Innovation and the Arts, Queensland, Australia; Biodiversity Insitute of Ontario – University of Guelph, Canada

## Abstract

Freshwater fish are a group that is especially susceptible to biodiversity loss as they often exist naturally in small, fragmented populations that are vulnerable to habitat degradation, pollution and introduction of exotic species. Relatively little is known about spatial dynamics of unperturbed populations of small-bodied freshwater fish species. This study examined population genetic structure of the purple spotted gudgeon (*Mogurnda adspersa*, Eleotridae), a small-bodied freshwater fish that is widely distributed in eastern Australia. The species is threatened in parts of its range but is common in coastal streams of central Queensland where this study took place. Microsatellite (msat) and mitochondrial DNA (mtDNA) variation was assessed for nine sites from four stream sections in two drainage basins. Very high levels of among population structure were observed (msat *F*_ST_ = 0.18; mtDNA Φ_ST_ = 0.85) and evidence for contemporary migration among populations was rare and limited to sites within the same section of stream. Hierarchical structuring of variation was best explained by stream section rather than by drainage basin. Estimates of contemporary effective population size for each site was low (range 28 – 63, Sibship method), but compared favorably with similar estimates for other freshwater fish species, and there was no genetic evidence for inbreeding or recent population bottlenecks. In conclusion, within a stable part of its range, *M adspersa* exists as a series of small, demographically stable populations that are highly isolated from one another. Complimentary patterns in microsatellites and mtDNA indicate this structuring is the result of long-term processes that have developed over a remarkably small spatial scale. High population structure and limited dispersal mean that recolonisation of locally extinct populations is only likely to occur from closely situated populations within stream sections. Limited potential for recolonisation should be considered as an important factor in conservation and management of this species.

## Introduction

Biodiversity is being lost in freshwaters at a faster rate than in any other environment [1], [2]. Approximately 40% of North America's freshwater fishes are either at risk or already lost, and estimates of future losses suggest that freshwater species are being lost five times faster than terrestrial species [1], [3]. Australia has a relatively depauperate freshwater fish fauna [4], so it is especially important to understand the processes responsible for maintaining populations and those that may be likely to pose a threat for their viability.

For those species that inhabit freshwater systems in the drier parts of the Australian continent (which represents about 75% of the total, [5]), populations often spend much of the year in waterholes that are isolated from one another because the river connecting them has ceased to flow [6]. Added to this, during periods of drought, it is likely that some of these rivers may not flow for a number of seasons, a situation that is exacerbated by human extraction of water for agricultural and urban water use. This can result in some isolated waterholes drying up and thus local populations of freshwater fish experiencing temporary extinctions [7]. Furthermore, the numbers of individuals in some of these water holes may become so low as to be unsustainable. Their effective population sizes may be so low that they lose most of their genetic diversity and inbreeding is common. These conditions are likely to threaten the viability of individual populations as they may be more susceptible to disease and to inbreeding depression [8]. The ability of a species to recolonise these habitats once flow is resumed will depend on a number of factors, the most important of which are probably the dispersal ability of the species and the degree of geographic/environmental isolation of the habitat. For species that have survived in these habitats over evolutionary time, it would be expected that they should have extremely good dispersal abilities, in order to make use of the opportunities to recolonise these habitats when they arise. This has been shown to be the case for a number of fish species that occur in dryland rivers of inland Australia [7], [9], [10]. For species that occur in regions that have more predictable environmental flow regimes and where drying and isolation have been less widespread, it is likely that selection for good dispersal ability has been less intense. Recently, however, large areas of eastern Australia have been subjected to long droughts and extensive water extraction, so that habitats that were once connected every season, may have become isolated for a number of years and drying out may cause local extinctions for populations throughout a sub-catchment, further reducing the potential source populations for recolonising these habitats.

The Murray-Darling Basin is by far the largest river system in Australia covering about a million square kilometers. It is also one of the most heavily regulated and used [11]. In recent years, a number of obligate freshwater fish have become rare or threatened in the system [12]. One such species is the purple-spotted gudgeon *Mogurnda adspersa*. This species was historically widespread throughout the Murray-Darling, as well as in coastal drainages of eastern Australia from Cape York Peninsula to northern New South Wales [13], [14]. The species has experienced severe declines in southern parts of its range including the Murray-Darling Basin since the 1980s and is now listed as endangered under the New South Wales fisheries Management Act 1994 [15]. Its decline has been suggested to be caused by the presence of introduced fish species [16] and loss of favourable habitat [17]. However, a more important driver may be that local extinctions have not been naturally recolonised from further afield, because the species does not have the necessary dispersal ability. Movement biology of *M. adspersa* is poorly understood [18]. A small-scale mark-recapture study demonstrated that short distance dispersal among pools within a 1 km creek segment is common [19]. However the ability of *M. adspersa* to disperse at the scale of the stream network is unknown and this larger scale is relevant for assessing the recolonisation potential of the species [20]. The Queensland government has used *Mogurnda adspersa* as an ‘ecological asset’ for Water Resource Plans (Queensland Water Act, 2000) in catchments where the species occurs. This is because the spawning success of *M. adspersa* depends upon stable, low-flow velocity habitat during the summer breeding season [18] and the availability of these conditions is determined by hydrology. This means that opportunities for spawning success and therefore population viability are vulnerable to alterations to river flow regimes by water resource development. Ecological models based on these relationships are used to assess the risk posed to *M. adspersa* population viability by alternative water management options and this assessment informs decisions about water management in the relevant Water Resource Plan. Effective use of this species as an ecological indicator in water resource planning and effective conservation of the species in southern areas where population declines and extinctions have occurred both require information on rates of dispersal among populations which can be obtained using genetic data (*e.g*. [21]).

There have been two phylogeographic studies of *M. adspersa* in eastern Australia. Hurwood and Hughes [22] examined this species in north-eastern Australia, using mitochondrial DNA (mtDNA) and found evidence for some connectivity across contemporary drainage divides, which was attributed to changes in the drainages themselves, rather than a good dispersal ability of the species. Nevertheless, there was still substantial genetic differentiation, when they recalculated levels of divergence between samples within the hypothesised historical drainages. Many of the sample sizes were very small however, so extrapolating these data to infer a general dispersal ability for the species would be questionable. Faulks *et al.* [15] also used mtDNA in a phylogeographic study on a broader scale, including data from Hurwood and Hughes [22] as well as additional samples from south-east Queensland and the Murray-Darling Basin, where the species is listed as endangered. They also found high levels of differentiation between their samples. They suggested that this supported the idea that the species was not a good disperser. Their study however was at a very broad scale, across the whole of the northern section of the Murray-Darling Basin and did not address the question of dispersal at the level of among reaches within a river. Furthermore, it too was based solely on mtDNA, which is more informative about historical dispersal patterns rather than contemporary gene flow [23].

The Pioneer River flows into the Pacific Ocean in central Queensland. The climate is tropical, with high rainfall, which occurs mostly in the summer months. Unlike in sub-catchments of the Murray-Darling system, *M. adspersa* is relatively abundant in this river and adjacent coastal streams. Thus, the aim of this study was to examine the relative isolation of populations in different pools within the system and adjacent coastal streams, to determine the level of dispersal between populations at a spatial scale relevant to recolonisation processes. We also wished to infer several demographic properties of *M. adspersa* populations that may be representative of the species in a non-threatened part of its range and will facilitate future comparison with threatened and/or re-established populations. These measures include effective population size (*N_e_*), the inbreeding coefficient (*F*_IS_) and evidence for recent population bottlenecks. Each of these measures is commonly used to evaluate the effect of small population size on patterns of neutral genetic variation in conservation genetic studies [8].

We used sequence data from mtDNA ATPase 6 and 8 genes combined with genotype data from 11 microsatellite loci to test the prediction that genetic structuring of *M. adspersa* populations would be consistent with limited dispersal within and between streams. We also predicted that subdivided populations in freshwater stream habitats would correspond with low effective population sizes and exhibit genetic evidence for inbreeding and population bottlenecks.

## Methods

### Sampling and study sites

**Figure 1:**
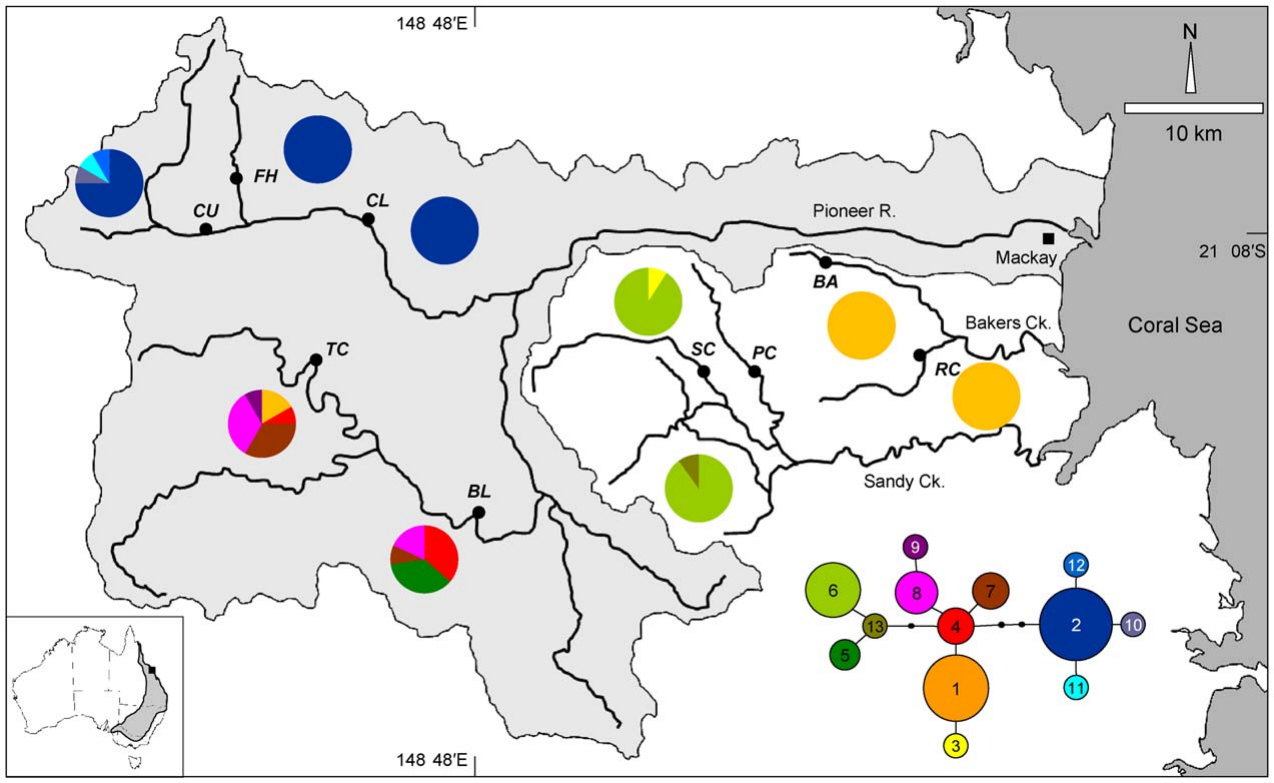
Map of study area. Location of study sites. Shaded area delimits the Pioneer River Basin boundary; Bakers Creek and Sandy Creek represent the Plane Basin. Inset map shows the geographic distribution of *Mogurnda adspersa* in Australia and position of the study area. mtDNA haplotype network shows the genealogical relationship among 13 haplotypes with pie charts illustrating the frequency of individual haplotypes within each sample site.

Sampling was undertaken during April, 2008 in the upper reaches of several adjacent streams in two coastal drainage basins of central Queensland. Between 16 and 81 fish were sampled from each of five sites in two sub-catchments of the Pioneer River basin and two sites in each of two coastal streams of the Plane basin (Table 1; Figure 1). Fish were sampled using electro-fishing and a small (2 mm^2^) tissue sample was excised from the caudal fin and stored in 100% ethanol before release at the point of capture.

**Table 1:** Sample information.

Basin name	Creek name	Site Code	Latitude (S)	Longitude (E)	Elevation (m)	Sample Size	*H*_O_	*H*_E_	*F*_IS_ (P)	Bottleneck
Pioneer	Finch Hatton	*FH*	21°06′49′′	148°38′04′′	105	30	0.603 (0.218)	0.593 (0.214)	-0.017 (NS)	0.793
Pioneer	Cattle	*CU*	21°08′11′′	148°37′28′′	111	31	0.613 (0.183)	0.605 (0.192)	-0.013 (NS)	0.861
Pioneer	Cattle	*CL*	21°08′03′′	148°43′32′′	74	78	0.637 (0.207)	0.654 (0.206)	0.027 (NS)	0.998
Pioneer	Blacks	*BL*	21°19′05′′	148°47′35′′	77	16	0.574 (0.211)	0.615 (0.190)	0.070 (NS)	0.959
Pioneer	Teemburra	*TC*	21°13′27′′	148°41′06′′	208	81	0.440 (0.256)	0.467 (0.280)	0.058 (NS)	0.990
Plane	Sandy	*SC*	21°14′31′′	148°57′41′′	34	55	0.630 (0.212)	0.637 (0.204)	0.011 (NS)	0.995
Plane	Perrys	*PC*	21°14′43′′	148°59′23′′	33	29	0.535 (0.274)	0.535 (0.271)	-0.001 (NS)	0.650
Plane	Rocky	*RC*	21°12′10′′	149°04′53′′	16	21	0.565 (0.224)	0.533 (0.213)	-0.061 (NS)	0.517
Plane	Bakers	*BA*	21°09′33′′	149°03′04′′	25	16	0.585 (0.267)	0.524 (0.199)	-0.121 (NS)	0.793

Study site locations, site codes, Inbreeding coefficient (*F*_IS_) and Bottleneck test results for nine study sites. *F*_IS_ for each site is averaged across eleven microsatellite loci and corresponding P-values refer to test for heterozygote excess. Observed heterozygosity (*H*_O_) and expected heterozygosity (*H*_E_) presented as mean across all loci with one standard deviation in parentheses. Results of Bottleneck analyses comprise P-values for one-tail Wilcoxon test for heterozygote excess. NS  =  not significant.

### Ethics Statement

All field and experimental protocols carried out in this study were approved by the Griffith University Animal Ethics Committee. All procedures were carried out according to Australian Ethics Committee protocol number ENV/12/08/AEC.

### Molecular Methods

Genomic DNA was extracted from fin tissue using a standard phenol-chloroform extraction [24]. A 950bp fragment of mitochondrial DNA spanning ATP synthase subunit 8 and ATP synthase subunit 6 genes (hereafter ATP) was amplified by polymerase chain reaction (PCR) using primers ATP8.2L8331 and COIII2H9236 (S. McCafferty, unpublished http://striweb.si.edu/bermingham/research/primers) and the protocol described by Woods *et al.* [25]. PCR product was enzymatically purified then sequenced using the Big Dye version 3.1 kit (Applied Biosystems). Sequencing was performed on an ABI 3130xl sequencer (Applied Biosystems) at Griffith University DNA sequencing facility. Sequences were edited and aligned with SEQUENCHER v4.9 (Gene Codes Corporation) and deposited in Genbank under accession numbers JN378815 – JN378827 (Table S1).

A total of 357 individuals were genotyped with eleven polymorphic microsatellite loci on an ABI 3130 sequencer (Applied Biosystems). Genotyped loci included 2mog1, 2mog2, 2mog3, 2mog4, 2mog6, 2mog9, 2mog10, 3mog3, 3mog6, 4mog2 and 4mog3, which were amplified using multi-tailed multiplexing methods described in Real *et al*. [26]. Data was scored using GENEMAPPER v4.0 (Applied Biosystems) and genotype frequencies were tested for null alleles, large allele drop-out and stuttering artifacts using MICRO-CHECKER v2.2.3 [27].

### Data analysis

#### Summary Statistics

Tests for linkage disequilibrium (LD) and departure of genotypic proportions from those expected under Hardy-Weinberg Equilibrium (HWE) for the microsatellite dataset were calculated with exact tests for each locus/population combination using default settings in GENEPOP v4.0 [28]. Genetic diversity statistics per sampling site (average gene diversity and mtDNA nucleotide diversity) were calculated using ARLEQUIN v3.5.1.2 [29]. The critical value (α) was corrected for multiple tests using the BY False Discovery Rate method (BY-FDR) which controls experiment-wide Type I error without the loss of power associated with the Bonferroni adjustment [30], [31].

#### Genetic Structure

We tested for the existence of distinct genetic groups in the set of individual multi-locus genotypes using a model-based Bayesian clustering method. The probability of an admixture model was tested for clusters (*K*) ranging from one to nine using STRUCTURE 2.3.1 [32]. Models were tested using eight independent MCMC simulations, each consisting of 1×10^6^ iterations after a burn-in of 5×10^5^ iterations. The most likely number of homogeneous clusters was assessed using the second-order rate of change L̋ (*K*), or Δ*K* following Evanno *et al*. [33], using the online application STRUCTURE HARVESTER [34].

Geographic structuring of genetic variation was assessed in ARLEQUIN v3.5.1.2 [29] by estimation of the fixation index *F*_ST_ using an analysis of molecular variance (AMOVA) framework. Estimates of *F*_ST_ were obtained for each locus separately and as a weighted average over the eleven microsatellite loci. For the mtDNA locus, the fixation index was calculated using haplotype frequency weighted by genetic distance among haplotypes (Φ_ST_). Statistical significance of *F*_ST_ and Φ_ST_ estimates were determined by 1000 permutations of individuals among populations. Hierarchical structuring of variation was tested using AMOVA in ARLEQUIN v3.5.1.2 [29], [35] according to stream connectivity and drainage division. Two hierarchical arrangements of the nine populations were analysed where the highest level was either a) drainage basin: sites divided into two drainage groups, Pioneer (FH, CU, CL, TC, BL) and Plane (SC, PC, BA, RC); or b) stream division: sites divided into four groups according connectivity of streams in the upper river reaches, Pioneer north (FH, CU, CL), Pioneer south (TC, BL), Sandy Ck. (SC, PC), Rocky Ck. (BA, RC). Three hierarchical levels of variation were tested for each arrangement, between groups (*F*_CT_), among sites within groups (*F*_SC_) and among all sites (*F*_ST_). For the mtDNA dataset, the analysis was conducted using both haplotype frequencies (*F*_ST_) and haplotype frequencies weighted by genetic distance (Φ_ST_). Genealogical relationships of mtDNA haplotypes sampled in the present study were estimated using statistical parsimony in TCS v1.21 [36].

#### Gene Flow

Contemporary migration rates (*m_ji_*, the proportion of immigrants in population *i* that arrive from population *j*) over the past few generations were estimated using a Bayesian assignment method implemented in the software BAYESASS v1.3 [37]. This method is based on the principle that immigrants and their progeny show temporary disequilibrium in their microsatellite genotypes relative to the focal population under the assumption that background migration rate is relatively low (*F*_ST_ >0.05) and that loci are in linkage equilibrium [38]. Analyses were run for 3×10^7^ iterations, sampling every 2000 iterations with a discarded burn-in of 10^7^ iterations. Delta values were adjusted to 0.12 to ensure that chain swapping occurred in approximately 50% of the total iterations as recommended by Wilson and Rannala [37] and the analysis was repeated six times with different random number seeds to evaluate consistency of results. Unidirectional estimates of *m_ji_* were made for all pairs of sites and rates were reported that fell outside the 95% confidence interval simulated for uninformative data [37].

Long-term (equilibrium) estimates of migration rate (*M_ji_*, immigration rate from population *j* to population *i* scaled by the mutation rate) were obtained using the software MIGRATE-N v3.1.6 [39]. As this method is concerned with estimation of migration over the longer term [40], we pooled sites within each of the four stream sections using the AMOVA grouping for stream division (*i.e.* Pioneer north  =  FH, CU, CL; Pioneer south  =  BL, TC; Sandy Ck  =  SC, PC; Bakers Ck  =  BA, RC) so that we could address the question of migration at this slightly larger scale and over a longer timeframe. This approach jointly estimates migration rate and effective population size using a coalescent model where the probability distribution of genealogies for the migration matrix is explored using a Markov Chain Monte Carlo (MCMC) search. Microsatellite mutation was modelled as a continuous Brownian process. The Bayesian implementation of MIGRATE-N involved an MCMC search of 50,000 burn-in steps followed by 2,000,000 steps with parameters recorded every 50 steps; a static heating scheme of four chains with temperatures (1.0, 1.3, 3.0, 10,000.0); uniform prior on theta (min:0.0, max:7.0, delta:0.7); uniform prior on migration (min:0.0, max:50.0, delta:5.0). MIGRATE was run six times with parameter values starting from *F*_ST_-based estimates and the distribution of parameter values was compared across runs to ensure overlap of 95% C.I. Effective sample size was >7000 for all parameters. For comparison with BAYESASS, *M_ji_* was converted to *m_ji_* using the equation: *m_ji_*  =  *M_ji_* × μ under the assumption that microsatellite mutation rate (μ) equals 5.0×10^−4^ per site per generation [41]. The parameter *m_ji_* can be interpreted as the proportion of population *i* that are immigrants arriving from population *j* each generation.

#### Effective Population Size, Bottleneck and Inbreeding Coefficient

A contemporary estimate of effective population size (*N*_e_) based on patterns of individual relatedness was obtained for each sampling site using the software COLONY v2.0 [42]. The sibship assignment method estimates *N*_e_ of the generation preceding the sample as a function of the frequencies of half and full siblings identified from multi-locus genotype data [43]. Under the assumption that each sample represents a single cohort of same-aged individuals, the *N*_e_ estimate produced by this method approximates the effective number of breeders (*N_b_*) in the preceding generation. Sibship assignment was implemented using the full-likelihood score option in COLONY v2.0. Replicate runs starting from different random number seeds indicated that *N*_e_ estimates were highly consistent across runs so a single analysis was performed for each sampling site.

A long-term (equilibrium) estimate of effective population size (*N*_e_) was obtained for each stream from the MIGRATE-N analysis as described above. The population-scaled mutation rate (Θ) was converted to *N*_e_ using the standard assumption that microsatellite mutation rate (μ) equals 5.0×10^−4^ per site per generation and substituting these values into the equation *N*_e_  =  Θ/4 μ.

Genetic evidence for a recent reduction in local population size was tested using the software BOTTLENECK v1.2.02 [44] which compares observed and expected heterozygosity where the expected value is the equilibrium expectation conditioned on the observed number of alleles [45]. This test is based on the principle that a recent reduction in effective population size will decrease the number of alleles faster than heterozygosity and produce significant discordance between the two estimates of heterozygosity [44]. The Two-Phased (TPM) mutation model was used, incorporating 95% single-step changes with variance of multiple-step changes set to 12%. Statistical significance was evaluated from 5000 simulations using the one-tailed Wilcoxon sign-rank test as recommended from the power analysis of Piry *et al*. [44]. The inbreeding coefficient (*F*_IS_) was calculated as an average over all microsatellite loci for each sample. Randomisation tests were used to determine whether populations suffered from a heterozygote deficit that would be expected to result from mating between close relatives within small populations. Observed *F*_IS_ values were compared to a null distribution for each population created by randomising alleles within individuals 2000 times using the software FSTAT v2.9.3 [46].

## Results

### Genetic Diversity

A total of 96 mtDNA sequences were obtained for the ATP region, the alignment was 670 bp in length, including 138 bp of the ATP subunit 8 gene and 532 bp of the ATP subunit 6 gene. There were 14 variable positions contributing to 13 haplotypes, which formed a shallow network spanning a maximum of seven parsimony steps (Figure 1). Haplotype and nucleotide diversity within sites varied from zero at four sites that were fixed for a single haplotype (FH, CL: Haplotype 2; BA, RC: Haplotype 1, Figure 1), up to ∼0.8 and ∼0.003 (haplotype and nucleotide diversity) for sites TC and BL in the southern branch of the Pioneer River (Figure 1, Table S1).

The genealogical network of mitochondrial haplotypes showed evidence of very strong spatial structure. One clade, consisting of haplotypes 2, 10, 11 and 12, and three parsimony steps from haplotype 4, occurred only in the north branch of the Pioneer River. Haplotypes in the southern branch of the Pioneer River also formed a closely related group, with the exception of haplotype 5 which was more closely related to the common haplotype in Sandy Creek. Bakers Creek had only a single haplotype, haplotype 1.

Tests for linkage disequilibrium (LD) revealed 26 cases of non-random association between genotypes (α = 0.05) from a total of 476 tests that could be performed. Correction for Type-I error using the FDR B-Y method (corrected α = 0.0074) reduced the number of significant LD comparisons to six, each involving a different pair of loci and indicating that the microsatellite dataset is not biased by linkage. The mean expected heterozygosity per population across eleven microsatellite loci ranged from 0.467 to 0.654 (Table 1). All loci were polymorphic in each population except at site TC where loci 2m1 and 2m3 were fixed for a single allele. Tests for Hardy-Weinberg Equilibrium (HWE) produced 18 significant deviations (α = 0.05) from a total of 96 tests, reducing to eight significant deviations after FDR B-Y correction (corrected α = 0.0097). The pattern of significant deviation was not consistent across loci or populations indicating that the dataset is free of systematic bias.

### Population Structure

Both microsatellites and mtDNA suggested very high levels of genetic structure from pairwise comparisons (Table 2). Based on microsatellites, all pairwise comparisons were statistically significant, even after correction for multiple tests. For mtDNA, there were a few non-significant comparisons, between the sites on the northern arm of the Pioneer and between the two sites within each of the two coastal creeks. However, in each case, there was virtually no variation, so power was very low.

**Table 2:** Pairwise estimates of *F*_ST_ for the microsatellite dataset (below diagonal) and Φ_ST_ for the mtDNA dataset (above diagonal).

	*FH*	*CU*	*CL*	*BL*	*TC*	*SC*	*PC*	*RC*	*BA*
*FH*	-	−0.037	0.000	**0.734**	**0.780**	**0.981**	**0.910**	**1.000**	**1.000**
*CU*	**0.069**	-	−0.016	**0.731**	**0.766**	**0.941**	**0.887**	**0.939**	**0.939**
*CL*	**0.058**	**0.061**	-	**0.757**	**0.799**	**0.983**	**0.919**	**1.000**	**1.000**
*BL*	**0.175**	**0.131**	**0.113**	-	0.160	**0.591**	**0.501**	**0.569**	**0.569**
*TC*	**0.272**	**0.213**	**0.204**	**0.100**	-	**0.761**	**0.676**	**0.541**	**0.541**
*SC*	**0.151**	**0.150**	**0.102**	**0.181**	**0.255**	-	−0.039	**0.976**	**0.976**
*PC*	**0.199**	**0.179**	**0.141**	**0.202**	**0.267**	**0.044**	-	**0.878**	**0.878**
*RC*	**0.199**	**0.159**	**0.153**	**0.179**	**0.227**	**0.112**	**0.123**	-	0.000
*BA*	**0.185**	**0.162**	**0.133**	**0.198**	**0.258**	**0.079**	**0.100**	**0.053**	-

Comparisons in bold were significantly differentiated after adjusting the critical value using the FDR B-Y correction (α = 0.5; adjusted critical value  = 0.012).

When sites were organised into a hierarchy with drainage basin as the highest level, in an AMOVA, both microsatellites and mtDNA suggested more differentiation among sites within basins than between the two basins (Table 3). However, when the analysis was repeated with stream division as the highest level in the hierarchy, both sets of markers indicated most variation was partitioned between streams, with less, but still significant differentiation among sites within streams.

**Table 3:** AMOVA for two hierarchical arrangements of the nine sample sites.

Source	mtDNA		microsatellites	
	% Variation	Fixation index	% Variation	Fixation index
a) Drainage basin				
Among basins	30.29	Φ_CT_ 0.303^NS^	6.26	*F*_CT_ 0.063***
Among sites within basins	54.51	Φ_SC_ 0.782***	12.61	*F*_SC_ 0.135***
Within sites	15.20	Φ_ST_ 0.848***	81.13	*F*_ST_ 0.189***
b) Stream division				
Among streams	83.31	Φ_CT_ 0.833***	12.55	*F*_CT_ 0.126***
Among sites within streams	1.49	Φ_SC_ 0.089*	5.58	*F*_SC_ 0.064***
Within sites	15.19	Φ_ST_ 0.848***	81.87	*F*_ST_ 0.181***

Hierarchical arrangement of sites: a) sites divided into two drainage basins: Pioneer and Plane; b) sites divided into four stream divisions, see Materials and Methods. NS  =  not significant; *P<0.05; ***P<0.001.

### Migration

Estimates of contemporary migration from the BAYESASS analysis supported findings from the population structure analysis, indicating that for seven of the nine sites, all individuals appeared to have come from that site, *i.e.* no immigration could be detected. The two exceptions were at PC, where a significant immigration rate of 0.29 (95% CI: 0.25, 0.33) was detected from nearby SC, and at RC where a significant immigration rate of 0.28 (95% CI: 0.21, 0.32) was detected from nearby BA. In both cases, this dispersal appeared to be in only one direction, *i.e*. from SC to PC and from BA to RC.

**Figure 2:**
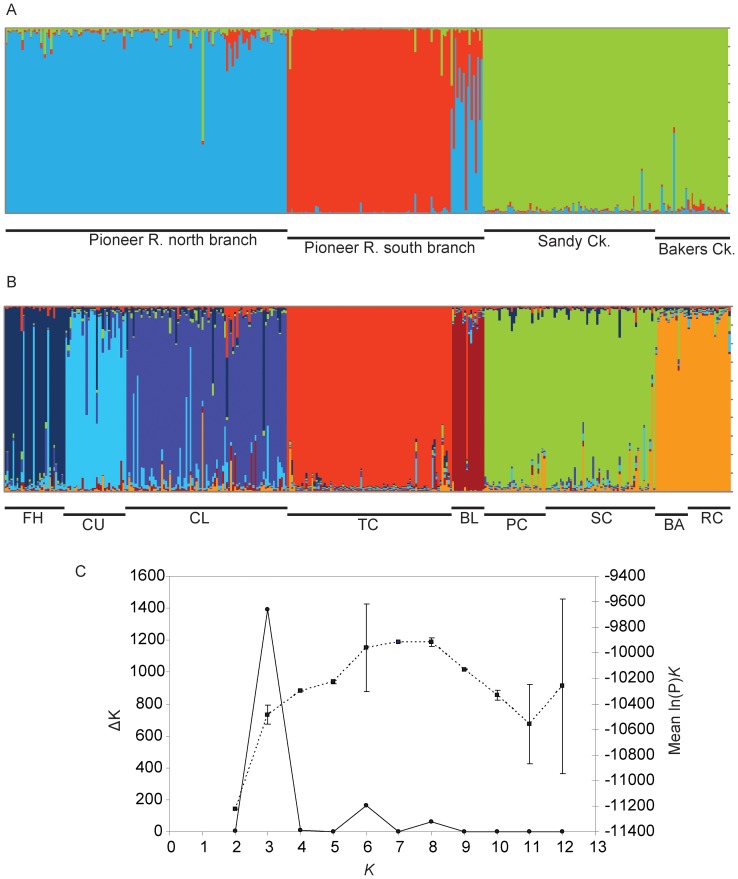
Population structure. Clustering analyses performed using STRUCTURE on multilocus microsatellite data. A) Bar plot of estimated membership coefficients (*q*) of each individual in *K* = 3 clusters. Black bars indicate the geographic location of individuals within the Pioneer and Plane basins. B) Bar plot of estimated membership of each individual in *K* = 7 clusters. Black bars indicate the location of individuals within the nine sample sites. C) Plot of Delta *K* (filled circles, solid line) calculated as the mean of the second-order rate of change in likelihood of *K* divided by the standard deviation of the likelihood of *K*, m(|L′′(*K*)|)/s [L(*K*)], also corresponding values for the mean likelihood (filled squares, dashed line) where the error bar represents one standard deviation.

Contemporary migration patterns were further elucidated by Bayesian clustering analysis using STRUCTURE. The highest mean likelihood score for the number of clusters (*K*) was seven (Figure 2c). Figure 2b shows the arrangement of these seven clusters in relation to individual fish grouped by sampling location. Fish from the five sites sampled in the Pioneer Basin (FH, CU, CL, BL, TC) were strongly structured and clearly correspond to five different clusters. Exceptions to this pattern include three individuals sampled from FH that were assigned with a high proportion of inferred ancestry (*q*>0.82) to the CU cluster, and one individual from BL that was assigned to the TC cluster (*q*>0.82; Figure 2b). These cases may represent contemporary immigrants that were not detected by the BAYESASS analysis. The two smaller coastal streams in the Plane Basin comprised the remaining two clusters, although sites within each stream ((SC, PC) and (BA, RC)) were not distinguished at all, suggesting that each of these small stream systems may represent a single population. This result was complemented by both the BAYEASS analysis which detected significant levels of contemporary migration between sites within each coastal stream (see above); and by analyses of population differentiation which showed that the lowest pairwise values of *F*_ST_ and Φ_ST_ were between sites within these coastal streams (Table 2). Application of the *ad hoc* Δ*K* approach to estimating *K* [33] showed that three clusters provide the best explanation for the uppermost hierarchical level of structure in the dataset (Figure 2c). Figure 2a shows the arrangement of these three clusters in relation to individual fish grouped by stream division (*i.e.* the northern and southern branches of the Pioneer River Basin and the two coastal creeks: Sandy Ck and Bakers Ck). Most individuals from the northern and southern branches of the Pioneer River were assigned to two distinct clusters (Figure 2a, blue and red bars), although individuals from site BL showed various degrees of admixture between these two clusters (*c.f.* Figure 2a, 2b). All individuals from Sandy Ck. and Bakers Ck. were assigned to the third cluster (Figure 2a, green bars). This result was complemented by the AMOVA analyses which showed that hierarchical partitioning of genetic variation was greatest between streams rather than between basins.

**Figure 3:**
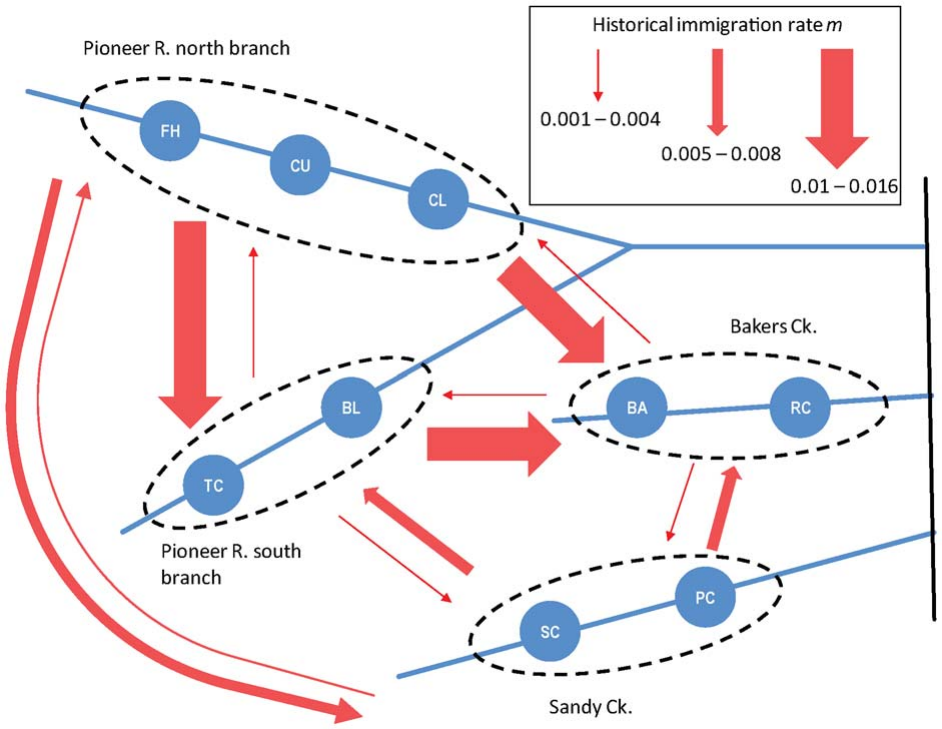
Summary of historical migration estimated among four stream sections. Immigration rate (*m*, the per generation probability that an individual is a migrant) based on multilocus microsatellite data and estimated using MIGRATE-N. Sample sites (blue circles) grouped into stream sections by dashed lines. Immigration rates between stream sections are illustrated by red arrows which represent direction of migration with arrow thickness divided into three size classes proportional to *m*.

Estimates of long-term migration among streams from MIGRATE-N showed historical migration has been low and asymmetrical. Among the four stream sections in this system the dominant source of immigrants appears to be the north branch of the Pioneer which showed a relatively high rate of migration into each of the other three stream sections (Figure 3). Even so, the estimated proportion of immigrants from the north Pioneer into each of the other streams per generation was very low (median estimates of *m* range from 0.008 to 0.016, Table S2). The lowest level of historical migration has been from Bakers Ck to the other streams, with Sandy Ck and the south arm of the Pioneer falling somewhere in between (Figure 3, Table S2).

### Population Size

**Figure 4:**
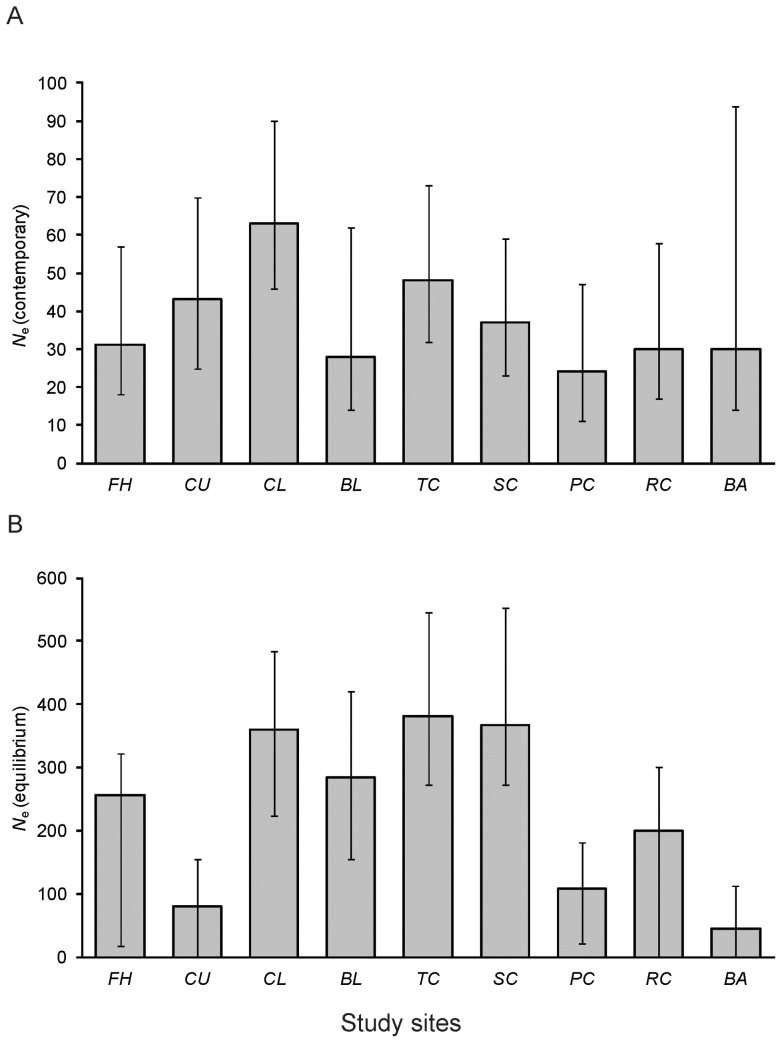
Estimates of effective population size. A) Contemporary effective population size (*N_e_*) for each sample site based on maximum likelihood estimation using COLONY software. Bar represents maximum likelihood *N_e_* estimate, error bars represent upper and lower 95% percentiles. B) Historical (equilibrium) effective population size for each sample site based on Bayesian inference using MIGRATE-N. Bar represents median *N_e_* estimate, error bars represent upper and lower 95% percentiles.

Because of the extremely high levels of population structure and indications of very little migration (both contemporary and historical) between sites, we estimated contemporary *N*_e_ values for each site separately using the Sibship method. All sites were estimated to have *N*_e_'s less than 100, with point estimates varying between 28 and 63 (Figure 4a, Table S3). Based on the credibility intervals, there did not appear to be different *N*_e_'s between populations, although the estimate for Bakers Ck had very large credibility intervals, possibly due to small sample size. Long-term equilibrium estimates of *N*_e_ from MIGRATE were higher, with point estimates for each site varying between 46 and 382 (Figure 4b, Table S3). Equilibrium estimates of *N*_e_ for the four stream sections (sites pooled) were quite variable and ranged from 140 for Bakers Ck to 1710 for the northern branch of the Pioneer (Table S4). The other two streams (southern Pioneer and Sandy Ck) had identical median estimates of *N*_e_ of 390 although different credibility intervals (Table S4). Evidently, the north branch of the Pioneer had a much larger *N*_e_ than the other streams and also historically has contributed more migrants to the other streams. No site showed evidence for a recent bottleneck as no significant excess in heterozygosity was found in any of the nine samples using Wilcoxon sign-rank tests (Table 1). Additionally, the average inbreeding coefficient (*F*_IS_) across loci for each of the nine sites ranged from −0.121 to 0.070 and none of these values corresponded with a significant heterozygote deficit expected under a scenario of inbreeding within small populations.

## Discussion

### Population Structure and Dispersal

Based on other studies, albeit only using mtDNA and on a larger geographic scale, we had predicted that we would find evidence that *M. adspersa* had limited dispersal abilities. We hypothesised that one explanation for the fact that the species has become threatened in parts of its range [15] is not only that the species has been unable to recolonise following localised extinctions, but also perhaps the effective size of individual populations is low, making them especially susceptible to effects associated with low genetic variation including inbreeding depression and reduced evolutionary potential.

Our results clearly support the notion that this species is a very poor disperser. All analyses suggested that dispersal among sites, even between those that are quite close and in the same segment of stream, was rare. The sites that we sampled were all within 20 – 30 km of another sampled site, yet, based on most analyses, there was no contemporary dispersal among the majority of them. The STRUCTURE analyses in particular, showed that each of the sites sampled in the Pioneer River appeared to be a separate population, with only a couple of examples of individual movement between proximate sites. The BAYESASS analysis was only able to detect contemporary movement of individuals between proximate sites within Sandy Ck and Bakers Ck. This evidence for highly restricted dispersal was expected given the results from mtDNA studies in other regions and at larger scales [15], [22]. In addition, the species appears to occur in pools, many of which are isolated from other pools by long stretches of unfavourable habitat. Our data therefore suggest that if local extinctions occur in one or more of these pools within a reach of the river, then recolonisation from elsewhere is unlikely to occur rapidly.

The results of this study show that patterns of genetic differentiation in both mtDNA and microsatellites in *M. adspersa* fit the Stream Hierarchy Model of Meffe and Vrijenhoek [47]. In other words, they reflect the dendritic structure of the stream with greater genetic differentiation among streams than among sites within streams. A similar pattern and level of structure has recently been reported for a closely related congener *M. mogurnda* in tropical northern Australia [48]. However, the levels of variation in the microsatellies in that study were considerably lower which tends to result in higher *F*_ST_ values, because less of the variation occurs within populations. Furthermore, in that study, both the geographic distances between sites and their elevation were substantially greater than in the present study. This further emphasizes the poor dispersal abilities of *M. adspersa*.

Very few studies have examined genetic structure of small-bodied freshwater fish in eastern Australian coastal streams and rivers, particularly at this relatively small scale. A study of the fly-specked hardyhead *Craterocephalus stercusmuscarum* in coastal drainages in north Queensland uncovered very high levels of divergence within drainages, but analysis of mtDNA suggested that the high and low altitude populations probably represented different historical colonisation events [49]. A similar study in the same area on another species, *Pseudomugil signifer*, showed that populations tended to fit an isolation by distance model, rather than the stream hierarchy model, although there were significant differences between populations within sub-catchments [50]. The spatial scale was similar to that in the present study and many of the pairwise comparisons of differentiation between sites within subcatchments were significant. Unfortunately, this study was based on allozymes and the analysis of data did not go beyond F-statistics, so it is not possible to determine if this species also shows as low a dispersal ability as *M. adspersa.* In terms of magnitude, there are several cases of freshwater fish that show higher levels of population genetic differentiation than *M. adspersa* over similar spatial scales. For example, a recent study of marble trout found a global *F*_ST_ of 0.716 for microsatellites [51], and pairwise microsatellite *F*_ST_'s up to 0.927 have been recorded for guppies [52]. However these values are for cases where populations have suffered recent perturbations or have highly asymmetrical migration patterns so the extreme structuring observed may reflect recent changes in genetic variability within populations. On the other hand, studies that show complimentary patterns of high structuring in both microsatellite and mtDNA markers are usually conducted over large spatial scales and incorporate multiple drainage divisions [*e.g*. 53,54,55]. Population genetic structure recorded here for *M. adspersa* is remarkable in that microsatellites and mtDNA both indicate strong complimentary patterns of differentiation that result from long-term processes, and have manifested over a relatively small spatial scale.

### Population Size and Viability

Estimates of effective population size based on the sibship method [42] indicated that all sites but one had *N_e_* less than 50 which appears low relative to suggested values of *N_e_*  = 50 to minimize inbreeding depression and *N_e_*  = 500 to maintain sufficient evolutionary potential [8]. However, comparison with *N_e_* values obtained using this method for other highly structured freshwater fish populations indicate that the *N_e_* values recovered for *M. adspersa* are not unusually low. For example, *N_e_* point estimates for three-spined stickleback ranged from 15 to 39 [56]; for brown trout the range was 16 to 32 [57]; and for the Tokyo bitterling the range was 5 to 28 [58]. The coalescent-based equilibrium *N_e_* estimation method [59] produced higher and more variable estimates, ranging from 46 to 382 for individual sites, and from 140 to 1710 for stream sections. Values of similar magnitude and similar levels of among site variation to these are typically found using this estimation method in other highly structured freshwater fish populations [52], [60]. Effective population sizes of *M. adspersa* samples measured in this study are therefore on par with those of other freshwater fish species. From a conservation genetics point of view, it seems unlikely that extinction risk for populations of *M. adspersa* is exacerbated by genetic stochasticity associated with small population size [61]. Lack of any genetic evidence for inbreeding or recent population bottlenecks is consistent with this conclusion.

Future studies of populations within threatened parts of the range of *M. adspersa* will be necessary to evaluate whether population declines are associated with the effects of genetic stochasticity. However it is unclear whether a general relationship between low effective population size, reduced fitness and elevated extinction risk should be assumed given the potential influence of factors including chance, selection and history [62]. For example, the effect of selection in purging the genetic load of small populations has experimental support in guppies [63] and this process may be associated with active inbreeding in wild populations of cichlid fish [64].

The different estimates of *N_e_* obtained using the two different methods applied here have been observed in other studies. As demonstrated by our results, coalescent-based equilibrium estimates of *N_e_* are generally higher than contemporary *N_e_* values obtained from short-term estimators. Fraser *et al*. [65] showed that equilibrium-based *N_e_* estimates were 2 – 10 times higher than short-term *N_e_* estimates in populations of trout and salmon. Potential explanations for this pattern are that populations have experienced declines at a recent point in their history, or that assumptions made by the equilibrium-based methods are violated such as overlapping generations or application of an incorrect mutation rate [65]. Eastern Australia experienced a prolonged period of drought over the ten years prior to sampling [66]. As a consequence it is possible that effective population sizes of these populations have declined recently. Even if this is the case, there was no evidence of recent bottlenecks, which suggests that although the *N_e_* values are generally low, they have probably been low for a significant time in the recent past. For values of *N_e_* around 50, the test should detect bottlenecks for 25–250 generations after the initial reduction in size [45]. Regardless of whether our results reflect recent declines, they clearly indicate a species with extremely limited dispersal capacity and a population structure consisting of a number of almost isolated populations with small effective population sizes. All these factors are likely to make this species very vulnerable to disturbance. Natural or anthropogenic changes to its particular low-flow hydrological requirements may challenge the resilience of *M. adspersa* given weak demographic links that exist among stream populations. The results support our idea that the specific life-history of this species has contributed to its significant reductions in distribution in the Murray-Darling system, where human disturbance and drought have significantly reduced hydrological connectivity among sites.

### Conservation Implications

While *M. adspersa* has experienced declines in the southern part of its distribution, it is still reasonably common in coastal Queensland rivers and streams. The reasons that the species has remained common in the northern part of its distribution could partly be due to the fact that extraction of water has less impact in these sub-tropical and tropical rivers, or alternatively, the impacts of human disturbance are yet to be realised. From our genetic analysis, we suggest that, although the species is currently apparently unaffected in many northern parts, further human disturbance may have serious impacts.

For example, Pusey *et al*. [67] have suggested that the introduction of sleepy cod into the Burdekin River has resulted in local extinctions of *M. adspersa* in that system.

Furthermore, the fact that the species is highly structured and appears to fit the Stream Hierarchy model allows us to predict the impacts of local extinctions at different spatial scales. If all populations in a single tributary are extirpated due to some spatially limited disturbance, such as pollution, then recolonisation of local sites is very unlikely. Alternatively, if a disturbance is not as severe, but more widespread across a catchment, such as a drought which causes some pools to dry out and others to remain as refugia, then recolonisation, although it may be slow, is more likely, as it can come from source populations in refugial pools within the same tributary.

While the effective population sizes estimated here are apparently low, it could be argued that, as a whole the *M. adspersa* in this region house significant genetic diversity. Effective population sizes could be calculated for the whole region, either by adding all the individual *N_e_* values or by calculating a pooled *N_e_*, as suggested by Palstra and Ruzzante [68]. When gene flow among populations is relatively high and structure is fairly low, the pooled *N_e_* value is similar to the sum of all the individual *N_e_*'s. However, when there are only moderate levels of structure, the pooled *N_e_* value is much reduced relative to the sum. In the case of these gudgeons, this presumably means that each time a local population is no longer viable, the extremely limited connectivity between populations will result in extinction with limited chance of recovery.

## Supporting Information

Table S1 mtDNA haplotype list. Haplotype and nucleotide diversity given for each site and Genbank accession number associated with each haplotype. Haplotype relationships are depicted in Figure 1.
(DOC)

Table S2 Immigration rate among four stream sections based on Bayesian analysis using MIGRATE-N. Sites pooled together into stream sections include: Section 1 (FH, CU, CL); Section 2 (BL, TC); Section 3 (SC, PC); Section 4 (RC, BA).
(DOC)

Table S3 Estimates of effective population size for each sample site. Values correspond with data presented in Figure 4.
(DOC)

Table S4 Estimates of effective population size for four stream sections. Sites pooled together into stream sections include: Section 1 (FH, CU, CL); Section 2 (BL, TC); Section 3 (SC, PC); Section 4 (RC, BA). Bayesian analysis using MIGRATE-N.
(DOC)
