# Supplementary material for: Extreme Genetic Structure in a Small-Bodied Freshwater Fish, the Purple Spotted Gudgeon, Mogurnda adspersa (Eleotridae)
Source: PLoS One. 2012 Jul 12;7(7):e40546. doi: 10.1371/journal.pone.0040546 (PMC3395642; doi:10.1371/journal.pone.0040546)
Supplement: Table S1 — mtDNA haplotype list. Haplotype and nucleotide diversity given for each site and Genbank accession number associated with each haplotype. Haplotype relationships are depicted in Figure 1. (DOC) [file pone.0040546.s001.doc]

|  |  | Site Code | | | | | | | | | |
| --- | --- | --- | --- | --- | --- | --- | --- | --- | --- | --- | --- |
| Haplotype  Code | Genbank Accession | *FH* | *CU* | *CL* | *TC* | *BL* | *PC* | *SC* | *BA* | *RC* | Total |
| Hap1 | JN378815 |  |  |  | 2 |  |  |  | 11 | 11 | 24 |
| Hap2 | JN378816 | 8 | 9 | 10 |  |  |  |  |  |  | 27 |
| Hap3 | JN378817 |  |  |  |  |  | 1 |  |  |  | 1 |
| Hap4 | JN378818 |  |  |  | 1 | 4 |  |  |  |  | 5 |
| Hap5 | JN378819 |  |  |  |  | 4 |  |  |  |  | 4 |
| Hap6 | JN378820 |  |  |  |  |  | 10 | 9 |  |  | 19 |
| Hap7 | JN378821 |  |  |  | 4 | 1 |  |  |  |  | 5 |
| Hap8 | JN378822 |  |  |  | 4 | 2 |  |  |  |  | 6 |
| Hap9 | JN378823 |  |  |  | 1 |  |  |  |  |  | 1 |
| Hap10 | JN378824 |  | 1 |  |  |  |  |  |  |  | 1 |
| Hap11 | JN378825 |  | 1 |  |  |  |  |  |  |  | 1 |
| Hap12 | JN378826 |  | 1 |  |  |  |  |  |  |  | 1 |
| Hap13 | JN378827 |  |  |  |  |  |  | 1 |  |  | 1 |
| Total |  | 8 | 12 | 10 | 12 | 11 | 11 | 10 | 11 | 11 |  |
| Haplotype diversity |  | 0 | 0.4545 | 0 | 0.8030 | 0.7636 | 0.1818 | 0.2000 | 0 | 0 |  |
| Nucleotide diversity |  | 0 | 0.0008 | 0 | 0.0022 | 0.0030 | 0.0014 | 0.0003 | 0 | 0 |  |

**Table S1 mtDNA haplotype list.** Haplotype and nucleotide diversity given for each site and Genbank accession number associated with each haplotype. Haplotype relationships are depicted in Figure 1.
