# Supplementary material for: Extreme Genetic Structure in a Small-Bodied Freshwater Fish, the Purple Spotted Gudgeon, Mogurnda adspersa (Eleotridae)
Source: PLoS One. 2012 Jul 12;7(7):e40546. doi: 10.1371/journal.pone.0040546 (PMC3395642; doi:10.1371/journal.pone.0040546)
Supplement: Table S2 — Immigration rate among four stream sections based on Bayesian analysis using MIGRATE-N. Sites pooled together into stream sections include: Section 1 (FH, CU, CL); Section 2 (BL, TC); Section 3 (SC, PC); Section 4 (RC, BA). (DOC) [file pone.0040546.s002.doc]

**Table S2 Immigration rate among four stream sections based on Bayesian analysis using MIGRATE-N.** Sites pooled together into stream sections include: Section 1 (FH, CU, CL); Section 2 (BL, TC); Section 3 (SC, PC); Section 4 (RC, BA).

| Immigration from section i to section j. | *M*_ij_ (immigration rate from i to j; scaled by mutation rate) | *m*_ij_ (immigration rate from i to j) | *m*_ij_ Lower 2.5% | *m*_ij_ Upper 97.5% |
| --- | --- | --- | --- | --- |
| m2-1 | 8.225 | 0.004113 | 0.003325 | 0.0049 |
| m3-1 | 5.075 | 0.002538 | 0.001692 | 0.003325 |
| m4-1 | 2.275 | 0.001138 | 0.00035 | 0.001867 |
| m1-2 | 24.908 | 0.012454 | 0.010909 | 0.013709 |
| m3-2 | 10.092 | 0.005046 | 0.0042 | 0.005775 |
| m4-2 | 3.442 | 0.001721 | 0.000934 | 0.00245 |
| m1-3 | 16.508 | 0.008254 | 0.00735 | 0.009159 |
| m2-3 | 7.525 | 0.003763 | 0.002742 | 0.004667 |
| m4-3 | 6.358 | 0.003179 | 0.002217 | 0.004025 |
| m1-4 | 32.025 | 0.016013 | 0.014759 | 0.017384 |
| m2-4 | 21.875 | 0.010938 | 0.009625 | 0.012659 |
| m3-4 | 14.292 | 0.007146 | 0.005834 | 0.008109 |
