# Supplementary material for: Extreme Genetic Structure in a Small-Bodied Freshwater Fish, the Purple Spotted Gudgeon, Mogurnda adspersa (Eleotridae)
Source: PLoS One. 2012 Jul 12;7(7):e40546. doi: 10.1371/journal.pone.0040546 (PMC3395642; doi:10.1371/journal.pone.0040546)
Supplement: Table S3 — Estimates of effective population size for each sample site. Values correspond with data presented in Figure 4. (DOC) [file pone.0040546.s003.doc]

**Table S3. Estimates of effective population size for each sample site.** Values correspond with data presented in Figure 4.

| Site Code | Equilibrium *N_e_*: MIGRATE-N method | | | Contemporary *N_e_*: Sibship method | | |
| --- | --- | --- | --- | --- | --- | --- |
|  | *N_e_* | Lower 2.5% | Upper 97.5% | *N_e_* | Lower 2.5% | Upper 97.5% |
| *FH* | 255.5 | 17.5 | 322 | 31 | 57 | 18 |
| *CU* | 80.5 | 0 | 154 | 43 | 70 | 25 |
| *CL* | 360.5 | 224 | 483 | 63 | 27 | 19 |
| *BL* | 283.5 | 154 | 420 | 28 | 62 | 14 |
| *TC* | 381.5 | 273 | 546 | 48 | 73 | 32 |
| *SC* | 367.5 | 273 | 553 | 37 | 59 | 23 |
| *PC* | 108.5 | 21 | 182 | 24 | 21 | 11 |
| *RC* | 199.5 | 0 | 301 | 30 | 58 | 17 |
| *BA* | 45.5 | 0 | 112 | 30 | 94 | 14 |
