# Supplementary material for: Extreme Genetic Structure in a Small-Bodied Freshwater Fish, the Purple Spotted Gudgeon, Mogurnda adspersa (Eleotridae)
Source: PLoS One. 2012 Jul 12;7(7):e40546. doi: 10.1371/journal.pone.0040546 (PMC3395642; doi:10.1371/journal.pone.0040546)
Supplement: Table S4 — Estimates of effective population size for four stream sections. Sites pooled together into stream sections include: Section 1 (FH, CU, CL); Section 2 (BL, TC); Section 3 (SC, PC); Section 4 (RC, BA). Bayesian analysis using MIGRATE-N. (DOC) [file pone.0040546.s004.doc]

**Table S4. Estimates of effective population size for four stream sections.** Sites pooled together into stream sections include: Section 1 (FH, CU, CL); Section 2 (BL, TC); Section 3 (SC, PC); Section 4 (RC, BA). Bayesian analysis using MIGRATE-N.

| Stream Section | *N_e_* | Lower 2.5% | Upper 97.5% |
| --- | --- | --- | --- |
| 1 (FH, CU, CL) | 1710 | 1350 | 2135 |
| 2 (BL, TC) | 390 | 100 | 665 |
| 3 (SC, PC) | 390 | 185 | 585 |
| 4 (RC, BA) | 140 | 0 | 315 |
